# Capacity development in health systems and policy research: a survey of the Canadian context

**DOI:** 10.1186/1478-4505-12-9

**Published:** 2014-02-07

**Authors:** Agnes Grudniewicz, Lindsay Hedden, Seija Kromm, Ruth Lavergne, Matthew Menear, Saskia Sivananthan

**Affiliations:** 1 Institute of Health Policy, Management and Evaluation, University of Toronto, Health Sciences Building, 155 College Street, Suite 425, Toronto, Ontario M5T 3M6, Canada; 2 Li Ka Shing Knowledge Institute, St. Michael’s Hospital, 209 Victoria Street, Toronto, Ontario M5B 1V8, Canada; 3 UBC Centre for Health Services and Policy Research, 201 - 2206 East Mall, Vancouver, British Columbia V6T 1Z3, Canada; 4 Canadian Health Human Resources Network, University of Ottawa – Institute of Population Health, 1 Stewart St, Room 227, Ottawa, Ontario K1H 8M5, Canada; 5 School of Public Health, Research Centre of the Centre Hospitalier de l’Universté de Montréal, University of Montreal, 7101, avenue du Parc, 3rd floor, Montreal, Quebec H3N 1X9, Canada

**Keywords:** Capacity development, Health systems and policy research, Human resources, Training, Workforce

## Abstract

**Background:**

Over the past decade, substantial global investment has been made to support health systems and policy research (HSPR), with considerable resources allocated to training. In Canada, signs point to a larger and more highly skilled HSPR workforce, but little is known about whether growth in HSPR human resource capacity is aligned with investments in other research infrastructure, or what happens to HSPR graduates following training.

**Methods:**

We collected data from the Canadian Institutes of Health Research, Canada’s national health research funding agency, and the Canadian Association for Health Services and Policy Research on recent graduates in the HSPR workforce. We also surveyed 45 Canadian HSPR training programs to determine what information they collect on the career experiences of graduates.

**Results:**

No university programs are currently engaged in systematic follow-up. Collaborative training programs funded by the national health research funding agency report performing short-term mandated tracking activities, but whether and how data are used is unclear. No programs collected information about whether graduates were using skills obtained in training, though information collected by the national funding agency suggests a minority (<30%) of doctoral-level trainees moving on to academic careers.

**Conclusions:**

Significant investments have been made to increase HSPR capacity in Canada and around the world but no systematic attempts to evaluate the impact of these investments have been made. As a research community, we have the expertise and responsibility to evaluate our health research human resources and should strive to build a stronger knowledge base to inform future investment in HSPR research capacity.

## Background

Over the past decade, significant investments have been made globally to support health systems and policy research (HSPR) with the ultimate goal of contributing to high-quality, accessible, and sustainable health care systems [1,2]. These investments have taken multiple forms, supporting both training and development of an HSPR workforce, as well as investments in research infrastructure.

The Canadian HSPR community has grown and transformed as a result of these investments; signs now point to a larger, younger, and more highly skilled HSPR community [3]. Developments in infrastructure, encompassing supportive research environments and tools (such as accessible data) are less apparent. If growth in human resource capacity has outpaced development of such research infrastructure, Canada’s transformed HSPR workforce may not be well-positioned to achieve its potential. Careful tracking of career trajectories and research outputs is required to ensure that investments in human resource capacity are yielding the desired growth in high-quality, relevant research [1].

The importance of conducting research on health research systems is gaining recognition [4]. A prominent stream in HSPR is the examination of health human resource capacity, which explores whether the health care workforce is meeting the needs of the population. This paper represents a first attempt to apply a similar lens to workforce issues facing the HSPR community in Canada.

### Trends in the broader scientific workforce

In other scientific disciplines, large increases in the number of graduates with advanced training have not been matched with growth in work opportunities that take advantage of trainees’ qualifications [5]. The number of completed science doctorates grew by 40% in OECD countries between 1998 and 2008, yet in the United States (US), the proportion of PhD graduates who received tenured academic positions within six years of completing their PhD fell from 55% in 1973 to 15% in 2006 [5]. Of course, unemployment among those with advanced degrees remains low, but the proportion of doctorates taking jobs that do not require a PhD is growing, as is the time it takes for graduates to find stable, rewarding positions [6]. This has broader implications, as it may signal that funding for training and scientific capacity development could be better directed. Whether these broad trends play out within HSPR is not known.

### Knowledge of the HSPR workforce

For the purpose of this study, we use the Canadian Institutes of Health Research (CIHR) definition of health services and policy research to define the broad field of health systems and policy research “as research designed to improve the way health care services are organized, regulated, managed, financed, paid for, used, and delivered” [1]. A 2007 AcademyHealth inventory of Health Services Research (HSR) training programs found 124 graduate programs in the US and Canada which graduate approximately 4,500 Master’s and 150–300 PhD students per year [7]. The US HSR field has grown from an estimated 5,000 health services researchers in 1995 to 11,600 in 2007 [8]. At the same time, US HSR funding declined (adjusted for inflation), pointing to a potential misalignment between workforce growth and opportunities for research funding and employment [9]. Knowledge of the HSPR workforce in the European context is even more limited as there is no Europe-wide HSPR society. Some countries do have professional HSPR organizations and national conferences [2,10]; however, this fragmentation hampers efforts to measure HSPR capacity globally.

Understanding the dynamics of the HSPR workforce is complicated by the diverse nature of the field, as researchers come from a variety of disciplines, collaborate internationally, and work in a wide range of settings [9,11]. US AcademyHealth membership indicates that nearly half of health services researchers were working in universities or teaching hospitals, one third in the private sector or foundations, and 10% in government agencies [8]. There is a noted lack of information on the HSPR workforce in Europe, where there are large differences in training opportunities among countries [10]. A report on HSPR capacity building in Canada (based on stakeholder interviews and a document review) concluded that good evidence is not available to capture Canada’s current HSPR human resource capacity; thus providing no information to direct further capacity building efforts [12]. To our knowledge, no Canadian studies have addressed this gap.

### Capacity development efforts in Canada

There have been considerable investments in the development of HSPR capacity in Canada over the past 10 to 15 years [3]. In 1999, the Canadian Health Services Research Foundation and the CIHR jointly launched the Capacity for Applied and Developmental Research and Evaluation (CADRE) program, a major 10-year capacity development initiative. CADRE supported mentoring chair awards, five Regional Training Centers (RTCs), post-doctoral awards, and career reorientation awards, with a total of $6.5 million in funding each year [13]. The RTCs provided students with the opportunity to collaborate with decision makers, gain an interdisciplinary perspective, and develop skills in research methods [13,14]. Shortly thereafter, in 2001, CIHR launched the $85 million Strategic Training in Health Research (STIHR) initiative to increase health research capacity through training and development of researchers, in health services as well as other areas [15].

**Figure 1:**
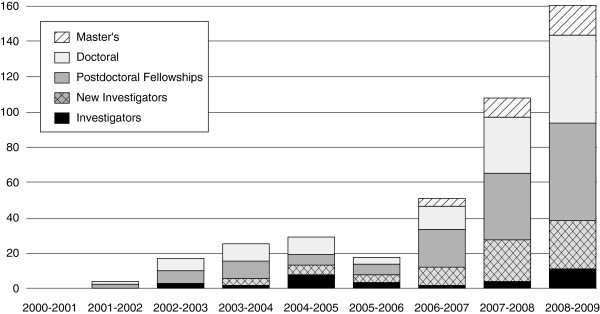
Number of health systems and services CIHR-funded training and salary awards, by year.

These direct investments in HSPR human resource capacity coincide with other policies that supported increased opportunities for advanced training. The establishment of CIHR’s Institute of Health Services and Policy Research in 2000 marked a profound change in the funding of HSPR research [16]. Prior to this, HSPR researchers were dependent on highly competitive fellowships funded through the Natural Sciences and Engineering Research Council of Canada. CIHR now provides targeted funding for student traineeships tied to operating grants, as well as master’s, doctoral, and post-doctoral awards. Figure 1 shows the marked increases in the number of master’s, doctoral, and post-doctoral awards, as well as more modest growth in new investigator salary awards. This figure does not capture research traineeships through operating grants, or through the RTC or STIHR programs.

Given these large investments in human resource capacity, we explored what information is collected on post-training activities of individuals who have recently graduated into the HSPR workforce in Canada. This is a first step in determining what is known, and what remains unknown, about the alignment between HSPR human resource capacity and research infrastructure.

Looking forward, we considered how we as both Canadian and international research communities might take steps to ensure that we fully capitalize on investments in human resource capacity.

## Methods

We collected data on recent graduates in the HSPR workforce from the CIHR, Canada’s national health research funding agency, and the Canadian Association of Health Services and Policy Research (CAHSPR), Canada’s national HSPR professional association. CIHR provided data on grant funding trajectories of previous doctoral award recipients. CAHSPR provided data on membership and annual conference registration.

These two data sources include only those young researchers who received CIHR doctoral funding or who were CAHSPR members or conference attendees, and are therefore incomplete in their ability to inform current workforce dynamics. To complement these sources, we conducted a survey of all Canadian university programs that provide HSPR training. We scanned Canadian university websites for programs that self-identified as providing graduate HSPR training, either via specific programs or within larger departments. The following were included: i) programs that offer research degrees in HSPR; ii) programs that offer research degrees in public, population, or community health, with options for HSPR specialization; and iii) graduate nursing programs that made specific reference to ‘health services or systems research’. Clinical fellowships with a research component or other MD prerequisite programs, executive programs, coursework-only degree programs, MBA or law specializations, information sciences, and public policy without explicit mention of health services or health systems were excluded. We then asked each of the identified programs/departments to confirm that they were in fact providing HSPR training, ensuring that we were only collecting data from relevant programs. A total of 45 programs were identified: 33 university-based programs and 12 collaborative and agency-based programs (RTCs and STIHRs). An additional file provides the list of HSPR training programs we included in our sample [Additional file 1].

We collected data between April and August of 2012. The email survey was sent to program directors and copied to assistant directors and program administrators where email addresses were publically available. Recipients were requested to forward the survey to the appropriate individual if misaddressed. The email included a short preamble introducing the CAHSPR Student Working Group, the survey objectives, and four open-ended questions (see below). The survey took less than ten minutes to complete and was sent in both English and French.

### The four survey open-ended questions

i.) Does [Program, School, or Department name] offer programs that provide training related to health services and policy research (HSPR)? If so, please list them.

HSPR has been defined by CIHR as research designed to improve the way health care services are organized, regulated, managed, financed, paid for, used, and delivered. Training could include a formal stream or certification, elective course offerings, or relevant research experience. Programs could include degrees (e.g., MPH, MSc, PhD), certificates, or fellowships.

ii.) How many students graduated from each identified program in the past three years (September 2008–November 2010)?

iii.) Have you collected information on the career experiences of previous graduates, following completion of these programs? For example, did they secure employment following graduation? If so, in what field (e.g., HSPR, public health, non-health-related) and sector (e.g., academia, health services delivery organization, government)?

If you have collected information, please describe in a few sentences the process you used, what types of information you gathered, and the years for which this was undertaken.

iv.) How many young investigators have been hired by your program within the past five years (July 2006–November 2010) who work in the area of HSPR?

A new investigator is defined as someone who has held a full-time research appointment for fewer than five years.

A reminder email was sent two weeks after the initial email request, followed by reminder phone calls to the program director or program administrator (extracted from contact information on program web pages) four, six, and eight weeks after the initial email request. When personal contact was not made, a voice mail message was left followed by another reminder email.

## Results

CIHR’s Institute of Health Services and Policy Research collects data on whether doctoral research award holders receive subsequent funding through grants. On average, 30% of graduated doctoral award holders receive grant funding and are assumed to be working in academia (in grant-tenured, tenure-track, or non-tenured academic positions). This figure rises to 71.4% among post-doctoral research award holders. Data from the CAHSPR conference reveal a young and growing community. Conference attendance grew from 443 in 2009 to 578 in 2012, with 35% of 2012 attendees registered as students. These data, however, provide a very limited picture of the HSPR workforce. There is no information about doctoral award winners who do not end up in academia, or trainees who did not receive CIHR awards. The question as to whether graduates are finding opportunities that use the skills they have acquired remains.

**Figure 2:**
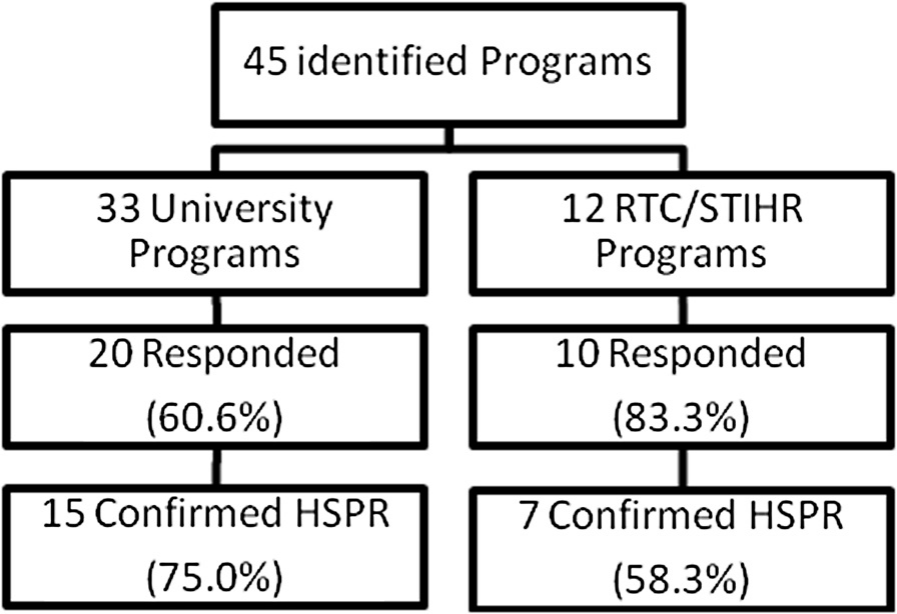
Survey responses from HSPR programs in Canada.

We sent our survey to 45 Canadian university and collaborative/agency-based programs. Of the 33 university programs contacted, we had a 61% response rate of which 75% confirmed provision of HSPR-specific training. Of the 12 collaborative and agency-based programs contacted, 83% responded, of which 58% confirmed HSPR training (Figure 2). Of these, three university programs and two RTC/STIHRs reported not having explicit HSPR programs, but provided support to students taking courses in HSPR and carrying out HSPR thesis research. Subsequent results focus on the 15 university and 7 RTC/STIHR programs that responded and confirmed they provided HSPR training.

The majority of university HSPR program responses were concentrated in Ontario, with a handful of programs in other provinces (Table 1). Most university programs (66.7%) graduated five or fewer HSPR students per year, likely because they are part of larger programs that offer some opportunity for HSPR specialization. In contrast, all but one RTC/STIHR program graduated more than five students per year, reflecting the focused HSPR training provided by RTC programs.

**Table 1:** Tracking activities of responding programs

	**University programs (n = 15)**	**RTC/STIHR (n = 7)**
	n (%)	n (%)
Program location*		
British Columbia	1 (6.7)	1 (14.3)
Alberta	1 (6.7)	0 (0.0)
Manitoba	0 (0.0)	0 (0.0)
Ontario	10 (66.7)	5 (71.4)
Quebec	2 (13.3)	1 (14.3)
Nova Scotia	1 (6.7)	0 (0.0)
Program size (graduates)**		
Small (≤ 5)	10 (66.7)	1 (14.3)
Medium (>5 and ≤ 20)	2 (13.3)	3 (42.9)
Large (>20)	2 (13.3)	2 (28.6)
**Tracking activities**		
One-time collection	1 (6.7)	3 (42.9)
Ongoing follow-up	2 (13.3)	4 (57.1)
None	12 (80.0)	0 (0.0)

*Of the university programs that did not respond, 3 were in British Columbia, 2 in Alberta, 1 in Saskatchewan, 1 in Manitoba, 3 in Ontario, 2 in Nova Scotia, and 1 in Newfoundland. Of the RTC/STIHRs that did not respond, 1 was in Quebec and 1 in the Maritimes. One STIHR supports training in Ontario and Western provinces.

**One university and one RTC program did not report the number of graduates over the past three years.

University programs do not consistently track students after graduation. Eighty percent do not track their graduates at all, providing only anecdotal examples of positions obtained (Table 1). All RTC/STIHR programs report tracking their graduate students, as mandated in their funding arrangements. Whether and how these data are used remains unclear. Three RTC/STIHR programs reported one-time data collection and four reported ongoing follow-up.

Only two (13.3%) university programs were able to provide information about their graduates’ careers (both graduating fewer than 5 trainees per year), compared to all seven RTC/STIHR program respondents. The university programs indicated that only nine (6.8%) of their recorded graduates obtained post-doctoral fellowships or research staff positions. Twenty-four (8.7%) of the 283 students who participated in RTC/STIHR programs were confirmed to be in HSPR faculty/teaching positions post-graduation. However, students in the RTC/STIHR programs come from a wide range of fields, and thus may be in faculty positions outside of HSPR.

University programs reported hiring a total of 7 young investigators that work in the area of HSPR over the period of July 2006 to November 2010. While this is not an accurate count of all academic hires of young HSPR investigators over this period, to our knowledge this information has not been tracked elsewhere. RTC/STIHR programs do not hire faculty or offer teaching positions.

No programs reported collecting information pertaining to whether their training had equipped graduates with the skills they needed post-graduation, nor assessed whether graduates were in positions that satisfied them or corresponded to their level of training.

## Discussion

The results of our survey demonstrate that most Canadian university programs do not follow their graduates in a systematic fashion, and data collected by relevant national organizations is limited. While RTC/STIHRs collected information on positions held by their graduates, there has been no reported examination of whether those graduates make full use of their training, or whether it has equipped them with relevant skills. There were also considerable challenges in comparing data across programs due to differences in definitions of participating students, as well as strategies and time frames for data collection. While a midterm review in 2008 reported that the RTCs were valued by both students and decision-makers, there were concerns about the quality of RTC’s tracking data [17]. A final evaluation of the RTCs has been commissioned, although at the time of writing of this paper, it had not yet been released.

Combining our survey results with the information collected by CAHSPR and CIHR, it is clear that there has been growth in HSPR training over the past decade, with a minority of trainees moving on to academic careers. However, much remains unknown about graduates’ career experiences and trajectories. As universities, funding bodies, government agencies, and research organizations have all played roles in the development of HSPR capacity in Canada and internationally, they also have roles to play in collecting information to inform this issue, and taking steps to ensure human resource capacity and research infrastructure are aligned.

Data on graduate employment would be useful to university departments and training programs as a tool for evaluating the quality of their programs and identifying opportunities for improvement. More specifically, these data could provide information on whether programs equip trainees with core competencies that match future career requirements [18]. Commenting on the scientific community more broadly, Kennedy et al. [6] suggest university departments give applicants a detailed account of the placement histories of recent graduates. As our results show, no departments surveyed currently possess this information. More careful tracking of graduates is a necessary first step. Research funders also have a role to play, ensuring that investments in human resource capacity are matched with support for research infrastructure, and ensuring that available support is balanced over the course of research careers. Support for new investigators has been identified as one possible gap in current career support funding [12]. While academic institutions are responsible for hiring faculty positions, models to support research infrastructure outside of the academic environment should also be explored.

Various factors may contribute to demand for HSPR and a need for growth in the field. In the US, demand for HSPR is expected to grow with large short-term funding increases resulting from the recent economic stimulus and health care reforms [9,19]. Expanding health data availability and complexity, the need for faster and more efficient knowledge translation, and pressures to cut costs in the health system may also continue to drive demand [7]. In Europe, the European Commission has funded ‘HSR Europe’ to identify research priorities in health services research, build health services research capacity, organize the health services research community, and define the relationship between research and policy [2]. It is also important to recognize that HSPR funding is imbalanced across countries, with low income countries further challenged by weak institutional capacity and a lack of critical mass within institutions [20]. Canada, and other high-income countries, may have a role to play in the development of the field internationally, in order to maximize global health system improvement [4].

While these forces may justify continued growth in Canada, to date, there is little information on current research capacity, let alone predictions of future needs. Better evidence will help to determine if continued investment in human resource development is warranted, or if resources could be better spent elsewhere in order to fulfill the objective of supporting high-quality, relevant research. Approaches to studying research capacity may be adapted from other jurisdictions to provide needed information on current research capacity and future needs specific to Canada [8,21,22].

Finally, we note that our study faces limitations inherent to self-report surveys. Though self-report surveys are not the ideal method of data collection, no other data was available to triangulate our results, and we believe this study is an important first step in understanding capacity development in Canada and identifying a lack of data collection on HSPR trainees.

## Conclusions

Over the past few decades, significant investments have been made to increase HSPR capacity worldwide, particularly in the area of training. Evidence suggests we now have many young, highly-trained students and new graduates. We know little, however, about the career trajectories of these students when they complete their advanced degrees. In Canada, university departments, training centers, and health research funders are only minimally engaged in student follow-up. A concerted and systematic, longitudinal effort to build a stronger knowledge base about HSPR capacity development is needed to inform future investment.

## Abbreviations

CADRE: Capacity for Applied and Developmental Research and Evaluation Program; CAHSPR: Canadian Association for Health Services and Policy Research; CIHR: Canadian Institutes of Health Research; HSPR: Health systems and policy research; HSR: Health services research; RTCs: Regional training centers; STIHR: Strategic training in health research.

## Competing interests

The authors declare that they have no competing interests.

## Authors’ contributions

AG conducted a review of the literature, drafted sections of the manuscript, commented on successive drafts, and prepared the final version. LH identified survey participants, drafted sections of the manuscript, and commented on successive drafts. SK collected and analyzed survey data, and commented on successive drafts. RL collected data from CIHR and CAHSPR, drafted sections of the manuscript, and commented on successive drafts. MM drafted sections of the manuscript and commented on successive drafts. SS collected and analyzed survey data, drafted sections of the manuscript, and commented on successive drafts. All authors contributed to the design of the study, conception of the manuscript, and approval of the final version.

## Authors’ information

The Canadian Association for Health Services and Policy Research (CAHSPR) Student Working Group contributing members (in alphabetical order): Agnes Grudniewicz, Institute of Health Policy, Management and Evaluation, University of Toronto & Li Ka Shing Knowledge Institute, St. Michael’s Hospital, Toronto, Canada. Lindsay Hedden, MSc, BSc (hons), Canadian Health Human Resources Network and Centre for Health Services and Policy Research, University of British Columbia, Vancouver, Canada. Seija Kromm, MA, BA (hons), BSc, Institute of Health Policy, Management and Evaluation, University of Toronto, Toronto, Canada. Ruth Lavergne, MSc, BSc (hons), Centre for Health Services and Policy Research, University of British Columbia, Vancouver, Canada. Matthew Menear, MSc, BSc (hons), School of Public Health, Research Centre of the Centre hospitalier de l’Universté de Montréal, University of Montreal, Montreal, Canada. Saskia Sivananthan, MSc, BSc, Centre for Health Services and Policy Research, University of British Columbia, Vancouver, Canada.

## Supplementary Material

Additional file 1 List of programs offering graduate HSPR training. Click here for file
