# Supplementary material for: Capacity development in health systems and policy research: a survey of the Canadian context
Source: Health Res Policy Syst. 2014 Feb 7;12:9. doi: 10.1186/1478-4505-12-9 (PMC3933033; doi:10.1186/1478-4505-12-9)
Supplement: Additional file 1 — List of programs offering graduate HSPR training. [file 1478-4505-12-9-S1.pdf]

Additional File 1: List of Programs Offering Graduate HSPR Training

University Training Programs

Division of Community Health and Humanities, Memorial University, Newfoundland

School of Health Administration, Dalhousie University, Nova Scotia

School of Nursing, Dalhousie University, Nova Scotia

Department of Community Health and Epidemiology, Dalhousie University, Nova Scotia

École des sciences infirmières, Université de Sherbrooke, Quebec

Département d'administration de la santé; Médecine sociale et préventive; Santé environnementale et santé au travail, Université de Montreal, Quebec

Department of Epidemiology, Biostatistics and Occupational Health, McGill University, Quebec

School of Nursing, McGill University, Quebec

School of Public Policy and Administration, Carleton University, Quebec

School of Nursing, University of Ottawa, Ontario

Population Health Program (PhD program), University of Ottawa, Ontario

Telfer School of Management (Health Systems), University of Ottawa, Ontario

Department of Epidemiology and Community Medicine, University of Ottawa, Ontario

Department of Community Health and Epidemiology, Queens University, Ontario

Department of Health Policy, Management, and Evaluation, University of Toronto, Ontario

Graduate Department of Rehabilitation Sciences, University of Toronto, Ontario

Graduate Department of Pharmacy, University of Toronto, Ontario

Graduate Department of Nursing Science, University of Toronto, Ontario

School of Health Policy and Management, York University, Ontario

School of Nursing, DeGroote School of Medicine, McMaster University, Ontario

Health Policy (PhD program), McMaster University, Ontario

Department of Epidemiology and Biostatistics, Waterloo University, Ontario

School of Nursing, Northern Ontario School of Medicine, Laurentian University

Department of Economics, Lakehead University, Ontario

School of Social Work, Lakehead University, Ontario

Department of Community Health Sciences, University of Manitoba, Manitoba

Department of Community Health and Epidemiology, University of Saskatchewan, Saskatchewan

Faculty of Nursing, University of Alberta, Alberta

School of Public Health, University of Alberta, Alberta

Department of Community Health Sciences, University of Calgary, Alberta

School of Population and Public Health, University of British Columbia, British Columbia

Faculty of Health Sciences, Simon Fraser University, British Columbia

Studies in Policy and Practice Program, , University of Victoria, British Columbia

Collaborative Programs

Western Regional Training Centre for Health Services Research

Ontario Training Centre in Health Services and Policy Research

Centre Ferasi

Atlantic Regional Training Centre - Health Services Research

CIHR-STIHR Training program in Health Law, Ethics & Policy

CIHR-STIHR Partnering in Community Health Research

CIHR-STIHR Programme de formation en analyse et évaluation des interventions en santé pour la prise de décision et les politiques (now AnÉIS)

CIHR-STIHR Integrated Training Program in Health and Social Science Research to Improve the Health of Marginalized Populations

CIHR-STIHR Ethics of Health Research and Policy

CIHR-STIHR Health Care, Technology and Place

CIHR-STIHR Transdisciplinary Understanding and Training on Research Primary Health Care (TUTOR-PHC)

CIHR-STIHR The Bridge Program
